# Systematic literature review and bibliometric analysis on virtual reality and education

**DOI:** 10.1007/s10639-022-11167-5

**Published:** 2022-06-27

**Authors:** Mario A. Rojas-Sánchez, Pedro R. Palos-Sánchez, José A. Folgado-Fernández

**Affiliations:** 1 Costa Rica Institute of Technology, Cartago, Costa Rica; 2 Department of Financial Economics and Operations, University of Sevilla, Seville, Spain; 3 NECE-UBI Research Unit in Business Sciences, University of Beira Interior (UBI), Covilhã, Portugal; 4 Departamento de Economía Financiera y Contabilidad, Universidad de Extremadura, Badajoz, España

**Keywords:** Virtual classroom, Learning management, Educational innovation, Virtual reality, Learning transfer, Educational technology, Learning processes, Bibliometrics

## Abstract

The objective of this study is to identify and analyze the scientific literature with a bibliometric analysis to find the main topics, authors, sources, most cited articles, and countries in the literature on virtual reality in education. Another aim is to understand the conceptual, intellectual, and social structure of the literature on the subject and identify the knowledge base of the use of VR in education and whether it is commonly used and integrated into teaching–learning processes. To do this, articles indexed in the Main Collections of the Web of Science, Scopus and Lens were analyzed for the period 2010 to 2021. The research results are presented in two parts: the first is a quantitative analysis that provides an overview of virtual reality (VR) technology used in the educational field, with tables, graphs, and maps, highlighting the main performance indicators for the production of articles and their citation. The results obtained found a total of 718 articles of which the following were analyzed 273 published articles. The second stage consisted of an inductive type of analysis that found six major groups in the cited articles, which are instruction and learning using VR, VR learning environments, use of VR in different fields of knowledge, learning processes using VR applications or games, learning processes employing simulation, and topics published during the Covid-19 pandemic. Another important aspect to mention is that VR is used in many different areas of education, but until the beginning of the pandemic the use of this so-called “disruptive process” came mainly from students, Institutions were reluctant and slow to accept and include VR in the teaching–learning processes.

## Introduction

The knowledge society recognizes that Education Institutions are a fundamental part of the globalization process, where the use of information and communication technologies (ICT) improve students' attitudes towards learning (Lazar & Panisoara, 2018). The concept of education refers to the process of facilitating learning, acquiring knowledge, skills or positive values with the aim of preparing students for life, work and citizenship (Kamińska et al., 2019). Virtual platforms often simulate the classroom and can provide a safe environment for testing experiments that can be dangerous in real life (Tzanavari & Tsapatsoulis, 2010). This revolutionizes learning processes, although professional training and scientific research are required, to facilitate innovative processes and develop new knowledge to meet the challenges of modern world demands (Castillo, 2010). The use of digital technologies has increased at all academic levels with educators adopting them in order to improve the learning experience of their students (McGovern et al., 2019).

Learning about sciences cannot always be fully implemented in classrooms, so it can be useful to use other options (Buehl, 2017; Folgado-Fernández et al., 2020). For example, scientific experiment in which physical risks would exist for students or use of very expensive scientific-technological material. In these cases, VR could simulate this environment and e-learning conditions. VR is suited to this as it consists of using a 3D environment that has been generated by a computer where the user can navigate and interact, achieving real-time simulation with a part, or all, of a user's senses (Guttentag, 2010). Another definition considers virtual reality as an immersive and interactive three-dimensional computer-generated environment in which interaction can occur on multiple sensory channels such as touch and position (Brey, 2014).

VR has been widely investigated in many fields such as sports (Vignais et al., 2015), tourism (Tussyadiah et al., 2018), virtual stores (Bonetti et al., 2018; Mann et al., 2015), healthcare from surgery simulation to nursing applications (Beyer-Berjot et al., 2016; Fagan et al., 2012), the military for flight simulation and training (Mihelj et al., 2014) and of course, in education (Zhang et al., 2017). Applications in education can be found for geography (Lv et al., 2017), nature sciences (Palos-Sanchez et al., 2022), mathematics (Xu & Ke, 2016), robotics (Román-Ibáñez et al., 2018), construction safety (Pham et al., 2018), medical training and assessment (Lövquist et al., 2012), physical education (Gómez-García et al., 2018) and many others.

Compared to traditional education, using virtual simulation-based training provides safety, cost savings and efficiency because less time is required for training (Shen et al., 2019a, b). Educators' acceptance of VR in the classroom has been successfully investigated by (Hussin et al., 2011). The behavioral intentions for using VR in learning was analyzed (Shen et al., 2019a, b) and also its acceptance by surgeons using VR for training (Hen, 2019).

The aim of this study is to identify and analyze the scientific literature with a bibliometric review to find the main topics, authors, sources, most cited articles and countries, as well as to know the conceptual, intellectual and social structure and identify the knowledge base of VR in education, and whether it is commonly used and integrated into teaching–learning processes. To reach the objectives, the articles in the scientific production indexed in the Web of Science and Scopus Main Collection were consulted, analyzing the articles and the emerging trends in research in articles published between January 1, 2010, and July 31, 2021.

This study analyzes relevant data from previous research to answer the following research questions (RQ) (Table 1):

**Table 1:** Research questions

	Research Question	Objective	Motivation
RQ1	Which authors and journals lead the literature on VR technology in education and which articles are cited the most?	To identify the most prolific sources and authors	To contribute to a better understanding of the scientific leadership in VR and Education
RQ2	What are the main topics that are researched, which countries contribute most to the scientific production and which words are most used in the literature on the use of VR technology in the educational field?	To show which topics are of most concern to researchers	To find out what topics scientific research is focusing on
RQ3	What are the bibliographic maps, graphs and tables for the data, along with the conceptual, intellectual and social structures and the knowledge base for the use of VR technology in the educational field?	To carry out an in-depth analysis and to represent it in a summarised form	To facilitate the understanding of the current situation of research in VR and Education
RQ4	What are the main research works related to VR and Education from an inductive analysis point of view?	To know the main works, methods applied, application and results obtained	To help the scientific community to improve its productivity

This article is organized as follows: the first part introduces the study, including the objectives and the research questions. A second section presents a review of the literature on bibliometric analysis. The methodology used is defined in the third section, indicating the search procedures used to identify the literature on VR in education. The fourth section presents the results, and these are discussed in the fifth section. Finally, in the sixth section, the conclusions are drawn, and future lines of research are suggested.

## Review of the literature on bibliometric analysis

This study uses a bibliometric analysis, which is a term that was coined by Pritchard (1969) who stated that it can be applied in all studies that aim to quantify the process of written communication (Gokhale et al., 2020).

Bibliometric analysis is an approach that uses a set of quantitative methods to measure, track, and analyze scholarly literature (Roemer & Borchardt, 2015). It identifies the publications by authors, the most prominent journals, as well as the methodologies used and the conclusions obtained (Durán Sánchez et al., 2014).

Metadata gives an overview of any field of research (Milian et al., 2019). Bibliometric methods involve a large volume of bibliographic material and have been used for the analysis of different topics (Blanco-Mesa et al., 2017), Journals (Martínez-López et al., 2018), Countries (Mas-Tur et al., 2019) and others.

The scientific literature contains important bibliometric analyses such as that by Huang et al. (2016), who performed a retrospective bibliometric analysis of articles about rehabilitation medicine using VR technology. The conclusion was that VR technology was one of the most popular technological advances. The results found a rapid growth in the production of articles in recent years.

## Methodology

In this study, the selected dataset is analyzed using a quantitative exploration with a bibliometric study that identifies and analyzes the literature on VR in education to provide a map of the knowledge structure (Álvarez-García et al., 2019). A performance analysis and scientific mapping is done in the first part of the study. The scientific or bibliometric mapping provides a representation of how disciplines, fields, specialties, individual papers, and authors are related to each other (Small, 1999). The recommendations of (Cobo et al., 2011) were used to produce the maps to know the research topics and the different structures in the dataset.

**Fig. 1:**
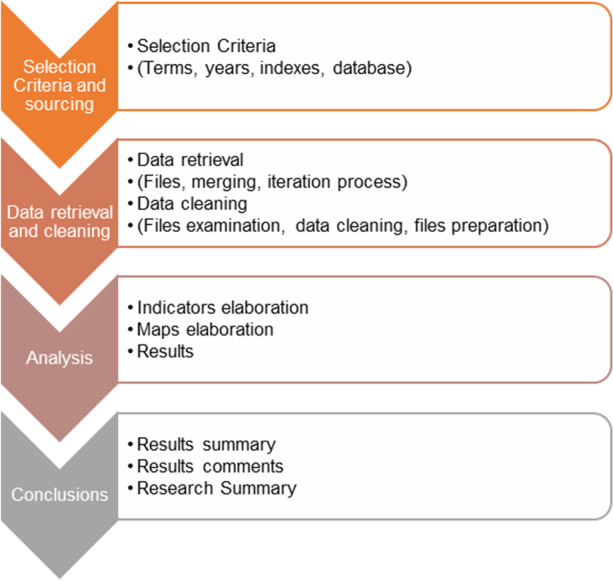
Methodology used. Methodology according to (Danvila-del-Valle et al., 2019)

A second analysis was also performed, ordering the articles in descending order according to the number of citations. The recommendations of (Heradio et al., 2016) were used to complete this part of the study. Figure 1 shows the methodology used. This figure shows different steps. For example, data cleaning was performed after applied inclusion criteria (see Table 2—Search string) or indicator elaboration permits sorted in relevance different articles.

**Table 2:** Documents obtained from searches in the databases

Data base	Search String	Results
WoS	TI = ((("virtual reality" and educat*)) or (("virtual reality" and learn*)) or (("virtual reality" and teach*)) or (("virtual reality" and class*)) or (("virtual reality" and student*)) or (("virtual reality" and innovat*)) or (("virtual reality" and covid*))) Refined by: Document Type: (Article), language: (English) and years of publication: 2010–2021(July). Exclude (Medicine)	436
Scopus:	(TITLE (“virtual reality" OR VR) AND TITLE (educat* OR learn* OR teach* OR class* OR student* OR innov* OR covid*)) Refined by: Document Type: (Article), language: (English) and years of publication: 2010–2021 (July). AND (EXCLUDE (SUBJAREA, “MEDI")	898
Lens:	((“virtual reality" OR VR AND educat*) OR (“virtual reality" OR VR AND learn*) OR (“virtual reality" OR VR AND teach*) OR (“virtual reality" OR VR AND class*) OR (“virtual reality" OR VR AND student*) OR (“virtual reality" OR VR AND innov*) OR (“virtual reality" OR VR AND covid)) Refined by Publication Type (Journal article), Field of Study (Virtual reality, Simulation, Teaching method, Higher education, immersive technology, Experiential learning, Knowledge management, Learning environment, Coronavirus disease 2019, Virtual learning environment, Google Cardboard, Training system, information technology)	1.161

### Dataset

Articles that constitute a representative sample of international scientific activity published in scientific journals were analyzed (Durán-Sánchez et al., 2018; Velasco et al., 2011). Therefore, meetings papers, editorials, books, chapters, proceedings, news, and other types of documents found in the databases were excluded.

### Identification of sources

Data was gathered from journal articles indexed in the Web of Science Core Collection. This database was selected because of three criteria:

- It has quality indexes such as JCR.
- It covers a long time period.
- It allows a considerable number of stored references to be downloaded simultaneously.

The presence of these characteristics is sufficient to justify its use (Durán-Sánchez et al., 2019).

The Scopus multidisciplinary bibliographic database was also used to find information in articles from scientific journals (ASJC) classified into an organized hierarchy of fields and subfields (Hassan et al., 2019). This database was selected because of three criteria:

- It has quality indexes such as SJR.
- It provides approximately 20% more coverage than the Web of Science.
- Simultaneous downloading of a considerable number of references is allowed (Falagas et al., 2008).

We used the Lens database and academic meta-search engine, compatible with the biblioshiny software used for data analysis. The search was limited to articles containing the keyword “virtual reality” in the title. This is included in quotation marks to obtain all documents containing that combination of words in the document title and also containing the possible combinations with the terms educat* (to obtain words that start with that word but may have different endings). The terms learn*, teach* class* and student* were also used to obtain articles with titles containing words related to learning, teaching, class or classroom, students. innovat* and Covid* were also included to obtain articles about innovation and the teaching process during the Covid pandemic.

### Study selection criteria

The search was performed in English to obtain the largest number of documents in the dataset on VR in education. Table 2 shows the initial data obtained with the proposed search strings. The inclusion criteria applied was document type: only articles were selected, language: English and years of publication: 2010–2021(July). The exclusion criteria were to exclude the field of Medicine. This study has not included specialized areas of medicine to get an overview of as many applications as possible in Education. It is particularly valuable for researchers, as information is presented about present and future lines of research which investigate the usefulness of VR in periods of crisis and confinement such as the one, we have just faced evidenced by the sudden leap in the use of technologies, and therefore virtuality, in platforms, applications, games and videos.

### Data analysis process

**Fig. 2:**
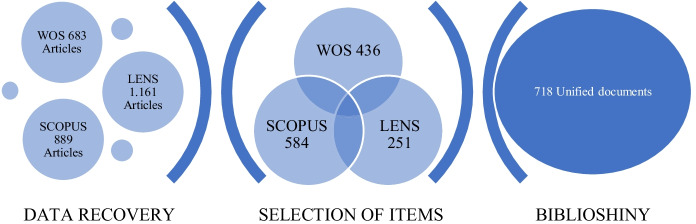
Search strategy summary. Note: Search strategy for the articles

The documents included in this analysis contain bibliographic information obtained after a manual review of the 436 relevant documents found in WoS, 584 found in Scopus and the 251 in Lens. 553 duplicate documents were eliminated, and the names of the authors and journals were normalized, which resulted in 718 documents unified in an.xlsx file. This whole process is summarised in the three stages of search strategies shown in Fig. 2.

Biblioshiny software was used for the analysis of the data. It is a tool which analyzes all the data identified in the body of literature and identifies the main themes (Huber, 2002). The application provides a web interface for the Bibliometrix software version 3.0 (Aria & Cuccurullo, 2017) and provides the data in graphical format, if desired, to visualize the statistics. In this study, the graphs describe the information about VR in the educational field over the time period chosen for the study. Figure 2 summarizes the search strategy used.

## Results

### SLR articles on VR and education

Tables 3 and 4 show some summarized systematic literature reviews (SLR) on virtual reality and education. There has recently been an increase in the number of documents, possibly due to current circumstances like the Covid pandemic and teleworking, which means that technological innovation in education systems has greater prominence than before.

**Table 3:** Summary of related systematic literature reviews

Systematic Literature Review Papers	Source	Period	Objective
Virtual reality in K‐12 and higher education: A systematic review of the literature from 2000 to 2019	Articles	2000–2019	To consolidate, evaluate, and communicate evidence that considers both the theory and practice of VR‐based instruction
Deep and Meaningful E-Learning with Social Virtual Reality Environments in Higher Education: A Systematic Literature Review (Mystakidis et al., 2021)	Peer-reviewed journals and conference proceedings	2004–2019	To find the effectiveness of e-learning along with the factors and conditions conducive to deep and meaningful learning, when using social virtual reality environments in distance learning higher education
Immersive virtual reality in K‐12 and higher education: A 10‐year systematic review of empirical research (Di Natale et al., 2020)	Articles	2010–2019	Mapping the use of Immersive Virtual Reality systems in K-12 and higher educational contexts and investigating their effectiveness in facilitating learning in terms of knowledge attainment and retention and motivational outcomes
A systematic review of immersive virtual reality applications for higher education: Design elements, lessons learned, and research agenda (Radianti et al., 2020)	Scientific journal articles and conference papers	2016 – 2018	To contribute to the existing body of knowledge about the application of digital devices for educational purposes
Virtual Reality and Computer Simulation in Social Work Education: A Systematic Review (Huttar & BrintzenhofeSzoc, 2020)	Articles and documents from annual meetings and conference programs	Oct. 2015 –Dec. 2016	To discover how social work as a profession has embraced virtual reality and computer simulation as instructive methods and their effectiveness in instruction
The emergence of technology in physical education: A general bibliometric analysis with a focus on virtual and augmented reality (Calabuig-Moreno et al., 2020)	Articles	1900 –2019	(1) To perform a bibliometric analysis of the articles published in the Web of Science on technology in PE (2) To analyze the articles published on augmented or virtual reality in PE found with this search
Virtual, augmented and mixed reality in K–12 education: a review of the literature (Maas & Hughes, 2020)	Peer-reviewed scholarly studies	2006 – May. 2017	Explore the peer-reviewed scholarly studies which address the use of virtual reality (VR), augmented reality (AR) or mixed reality (MR) technologies in the instruction of students in elementary, middle or high school

**Table 4:** Main information

Description	Results
Timespan	2010:2021
Sources (Journals, Books, etc.)	298
Average years since publication	2,38
Average citations per documents	10,03
Average citations per year per doc	2,489
References	10,955
Articles	718
Keywords Plus (ID)	1634
Author's Keywords (DE)	1700
Authors	1939
Author Appearances	2390
Authors of single-authored documents	72
Authors of multi-authored documents	1867
Single-authored documents	77
Documents per Author	0,339
Authors per Document	2,95
Co-Authors per Documents	3,64
Collaboration Index	3,22

Main statistical indicators

**Fig. 3:**
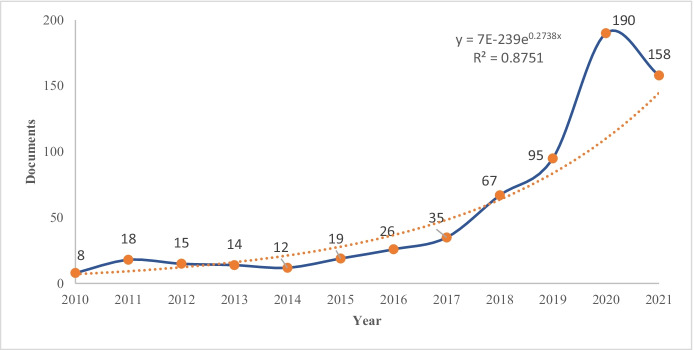
Annual production of articles. Note: Annual productivity of virtual reality in education. Dotted line is the exponential trend line

The use of ICTs in education is commonplace, with VR no exception and often included in the teaching–learning processes. The evolution in the productivity of articles over the period analyzed clearly shows a rapid growth from 2015 to 2021, as shown in Fig. 3.

This growth is due to the development of specific content in VR, with more and more sectors involved such as: real estate, locomotion, security, and even education itself with the new e-Learning systems.

### Performance analysis

According to Heradio et al. (2016) the main procedure for research performance evaluation is citation analysis, which means, the more citations of an article, the greater its influence in that field. The h-index is considered a suitable measure of the quantity and impact of the scientific output of the publications of a researcher.

#### Overview of the analyzed data set

The information from the analyzed data is summarized in descriptive statistics presented in Table 4. Considering the results obtained, we can say that RV is a topic of great academic interest as evidenced by the number of papers (718) and the more than ten average citations per article.

**Fig. 4:**
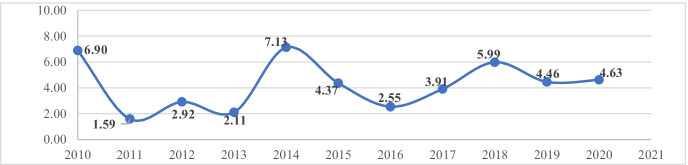
Average annual citations per year. Note: Average total number of citation per year

**Fig. 5:**
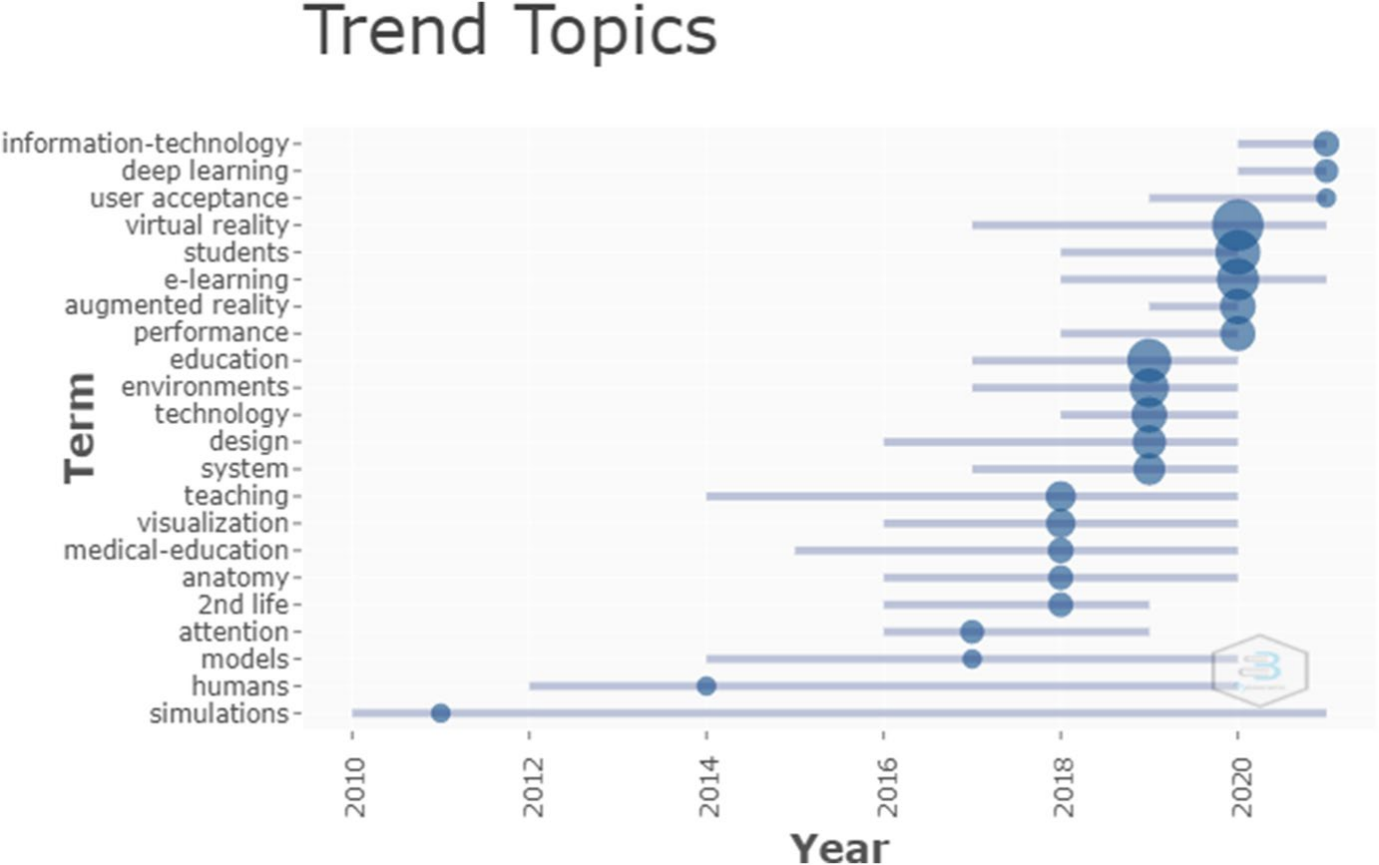
Trend tropics. Journals with most productivity and impact according to the h-index

The average number of annual citations are presented in Fig. 4, while Fig. 5 provides an overview of the trends in the knowledge structure of the use of VR in education.

Figure 4 shows that the highest average number of citations per year were in 2010 with 6.9 citations per year and in 2014 with 7.3. Contrary to what the authors expected due to the important advances and changes in the market for VR, there were only 2.55 citations in 2016 continuing with little growth until 2018 and maintaining lower averages thereafter. 2020 and the current, available data for 2021 does not show an impact on citations due to the covid-19 situation.

Figure 5 shows that the main topics of the trends from 2010 to 2017 were about using simulation as a learning tool to obtain greater student attention. 2018 continued integrating technology into the teaching process with topics such as the virtual community of players of 2nd life with customizable avatars that allow players to enjoy a second life. This uses voice text messaging with people from different places and countries and integrates visualization tools. In 2019 education included the design of technological environments. 2020 showed an increase in the literature consulted about e-learning using tools such as virtual or augmented reality. This could possibly be because of the changes in education methods imposed by the Covid-19 pandemic and the change from presential to virtual teaching, which was made abruptly in some cases. It should be noted that 6 months into 2021 the trend is towards platforms that can be used to teach or attend lessons, i.e., information technologies and user acceptance of these take on greater importance and there is also increasing interest in artificial intelligence with deep learning that uses machine learning processes such as speech recognition or automated translation.

The journal with the most impact in this study is Computers & Education with an h-index of 16. This means that a number, h, of publications of the journal have been cited h times. An h-index of 16 implies that this number of publications have been cited at least 16 times. Table 4 shows the journals ordered by the number of documents published, as well as the impact measured with the h-index. We can say that the selected journals contain 207 articles in total, of which 40% correspond to 3 publications: International Journal of Emerging Technologies in Learning, Computers & Education and Virtual Reality (Table 5).

**Table 5:** Magazines with most productivity and impact

Source	Articles	H-Index
International Journal of Emerging Technologies in Learning	30	8
Computers & Education	26	16
Virtual Reality	26	9
British Journal of Educational Technology	16	6
Interactive Learning Environments	16	8
Computer Applications in Engineering Education	12	6
Sustainability	11	5
IEEE Access	10	3
Multimedia Tools and Applications	10	4
Education and Information Technologies	9	3
International Journal of Interactive Mobile Technologies	8	4
Journal of Computers in Education	7	4
Educational Technology and Society	7	4
International Journal of Engineering Education	7	4
Australasian Journal of Educational Technology	6	5
Journal of computer assisted learning	6	4

Relevant information on the journals included in the dataset

#### Authors with most impact according to h-index

**Fig. 6:**
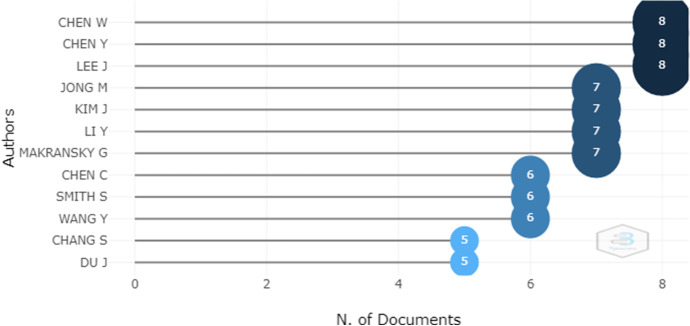
Most relevant authors

**Fig. 7:**
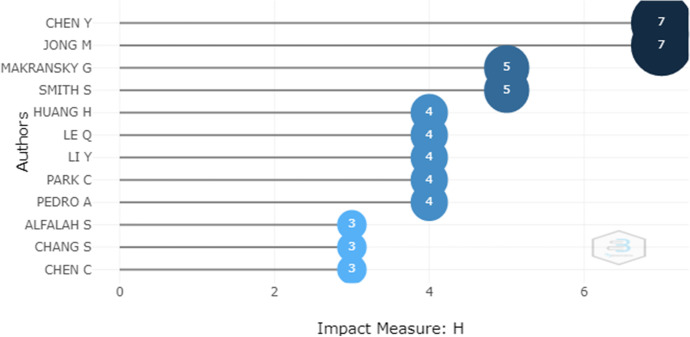
Impact of the authors

The authors with the highest productivity are shown in Fig. 6 and can be seen to be Chen W., Chen Y. and Lee J. Figure 6 orders the authors according to impact where it can be seen that Chen Y. and Jong M. have the highest impact with an h-index of 7, that is, each author has 7 papers with at least 7 citations each, which means that the author has been included in at least 49 publications (Fig. 7).

**Fig. 8:**
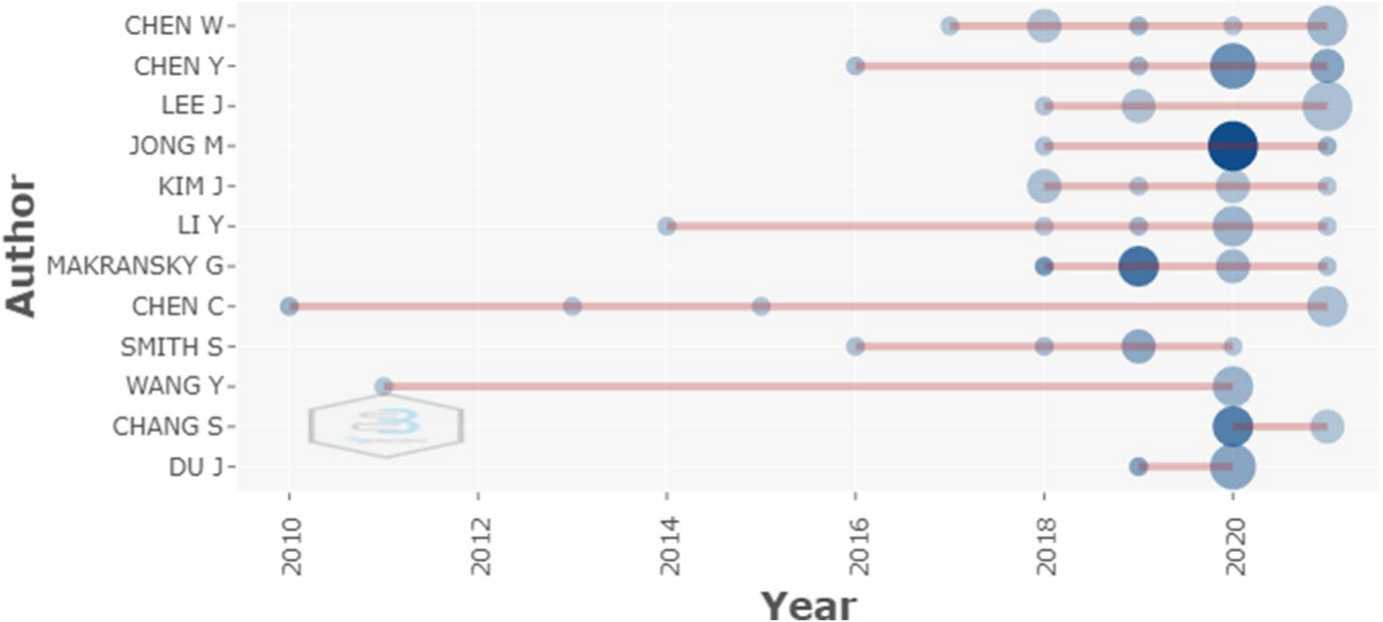
Productivity of the main authors over the period of time studied

The most active authors in the last four years have been Chen W., Lee J., Kim, J., Ly Y. and Makransky G., as shown in Fig. 8.

Chen W. primarily studies problem solving in the classroom using VR technology, to assist in cognitive processing and knowledge transfer to the students. On the other hand, Lee J., studies the adaptation of the three-dimensional visualization made possible by immersive virtual reality.

Other authors, such as Kim J., jointly approach the analysis of VR and augmented reality (AR) whith the current skin electronics are summarized as one of the most promising device solutions for future VR/AR devices.

Ly Yan approaches the study of VR based simulation in hospital settings, that facilitates the acquisition of skills without compromising patient safety. Finally, Makransky's research deals with various aspects of RV in education, such as an important role in education by increasing student engagement and motivation.

**Fig. 9:**
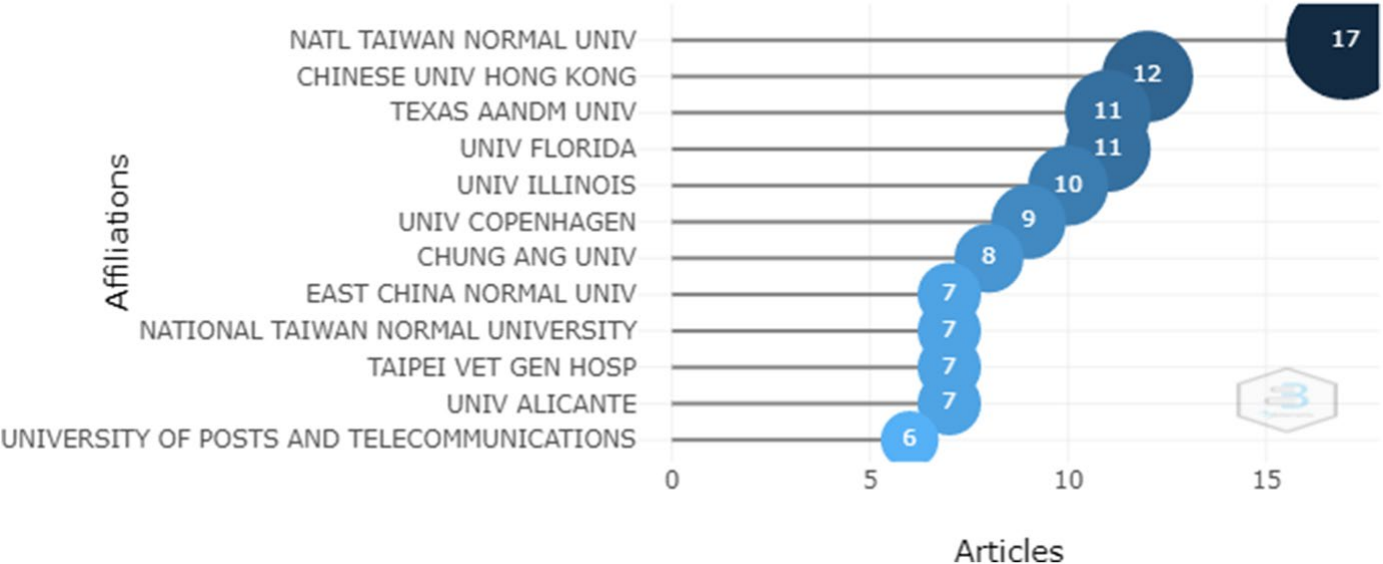
Most relevant affiliations

The main affiliations of institutions can be seen in Fig. 9, which shows National Taiwan Normal University as being the most productive with 17 papers published in the analyzed dataset. In second position is Chinese University Hong Kong with 12 papers and in third position is Texas Aandm University with 11 papers.

#### Main documents and most frequently used words in the dataset

Table 6 shows the documents with the largest number of citations in this study. The authors of the document with most citations were (Merchant et al., 2014) with 452 citations and, in second place, (Huang et al., 2010) with 236 citations, both of which were published in the Computer & Education journal. In third place was (Jensen & Konradsen, 2018) with 196 citations in the Telematics and Informatics journal. When an article has many citations, it influences the researchers who develop the area under investigation (Rodríguez & Navarro, 2008).

**Table 6:** Most globally cited documents in the dataset

Authors	Title	Source	Total citations	Highlight
Merchant et al. (2014)	Effectiveness of virtual reality-based instruction on students learning outcomes in k12 and higher education a meta-analysis	*Computers & Education*	452	A comprehensive review of virtual reality-based instruction research
Huang et al. (2010)	Investigating learners’ attitudes toward virtual reality learning environments: Based on a constructivist approach	*Computers & Education*	236	This paper introduces the educational use of Web-based 3D technologies and highlights VR features
Jensen and Konradsen, (2018)	A review of the use of virtual reality head-mounted displays in education and training	*Education and Information Technologies*	196	The review identified a number of situations where HMDs are useful for skills acquisition
Radianti et al. (2020)	A systematic review of immersive virtual reality applications for higher education: Design elements, lessons learned, and research agenda	*Computers & Education*	171	This study identifies 18 application domains VR, indicating a better reception of this technology in many disciplines
Lee et al. (2010)	How does desktop virtual reality enhance learning outcomes. A structural equation modeling approach	*Computers & Education*	159	The results show how to improve the learning effectiveness and their VR-based learning implementation
Merchant et al. (2012)	The learner characteristics features of desktop 3d virtual reality environments college chemistry instruction a structural equation modeling analysis	*Computers & Education*	104	Science achievements can be improved at the college level using 3D virtual reality
Fowler, (2015)	Virtual reality and learning: Where is the pedagogy?	*British Journal of Educational Technology*	103	The paper adopts a “design for learning” perspective, useful to those designing learning activities in 3-D VLEs

**Fig. 10:**
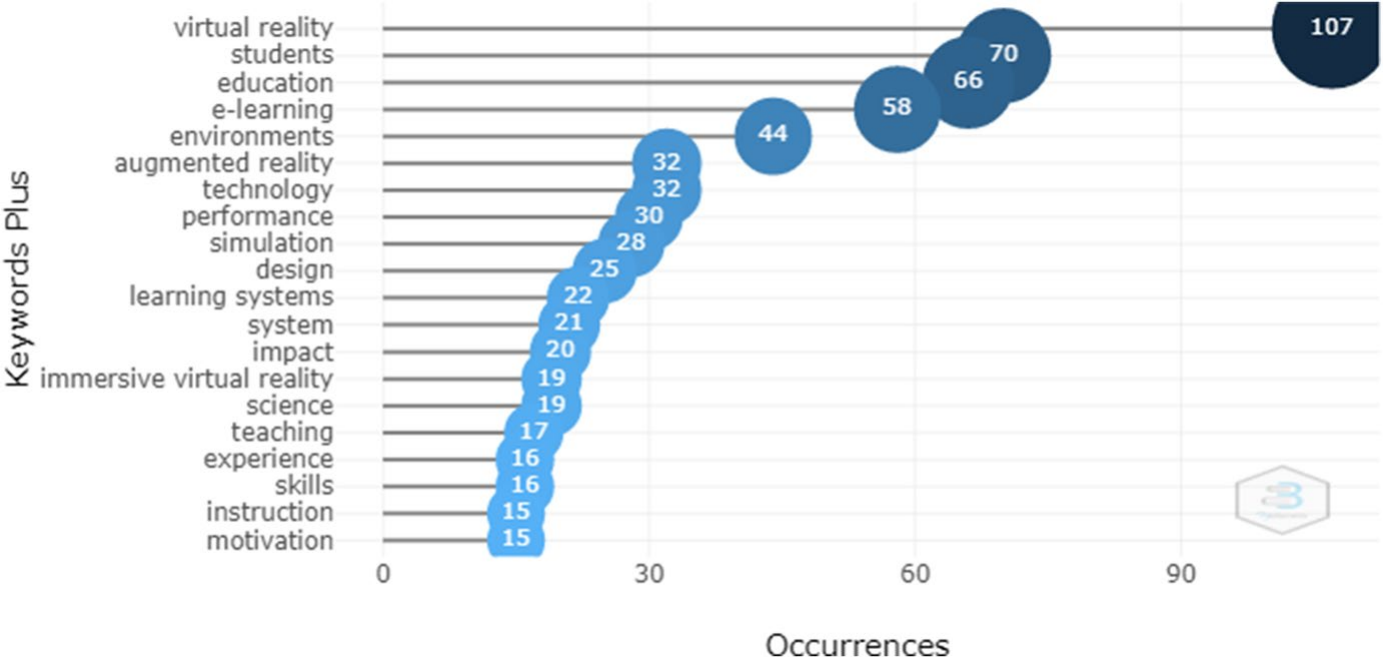
Most relevant words

The words which occur with the highest frequency in the dataset can be seen in Fig. 10. The first four words are related to the terms contained in the search strings, but the frequency and hierarchy follows the occurrences of the words “e-learning”, “environments”, “augmented reality”, “technology”, “simulation” and “learning systems”. This highlights the technological component of the field of education. The coincidence of keywords represents the knowledge structure of the literature (Cheng et al., 2018).

### Scientific mapping analysis

A similarity measure known as the strength of association was used to construct the bibliometric maps (Cobo et al., 2011; Van Eck & Waltman, 2007). This allows a variety of scientific maps to be prepared which show the structural and dynamic aspects of the data obtained from the scientific research (Börner et al., 2003).

According to Cobo et al. (2011) the maps show the evolution of a field of research and the conceptual structure of the field can be found from the co-occurrence. Co-citation and bibliographic coupling allow us to analyze the intellectual structure of a field of scientific research and the social structure can be found by analyzing the authors, also known as co-authorship analysis, as well as the data found from the author's affiliations such as the organization or country.

#### Main topics in keywords plus according to factor analysis

**Fig. 11:**
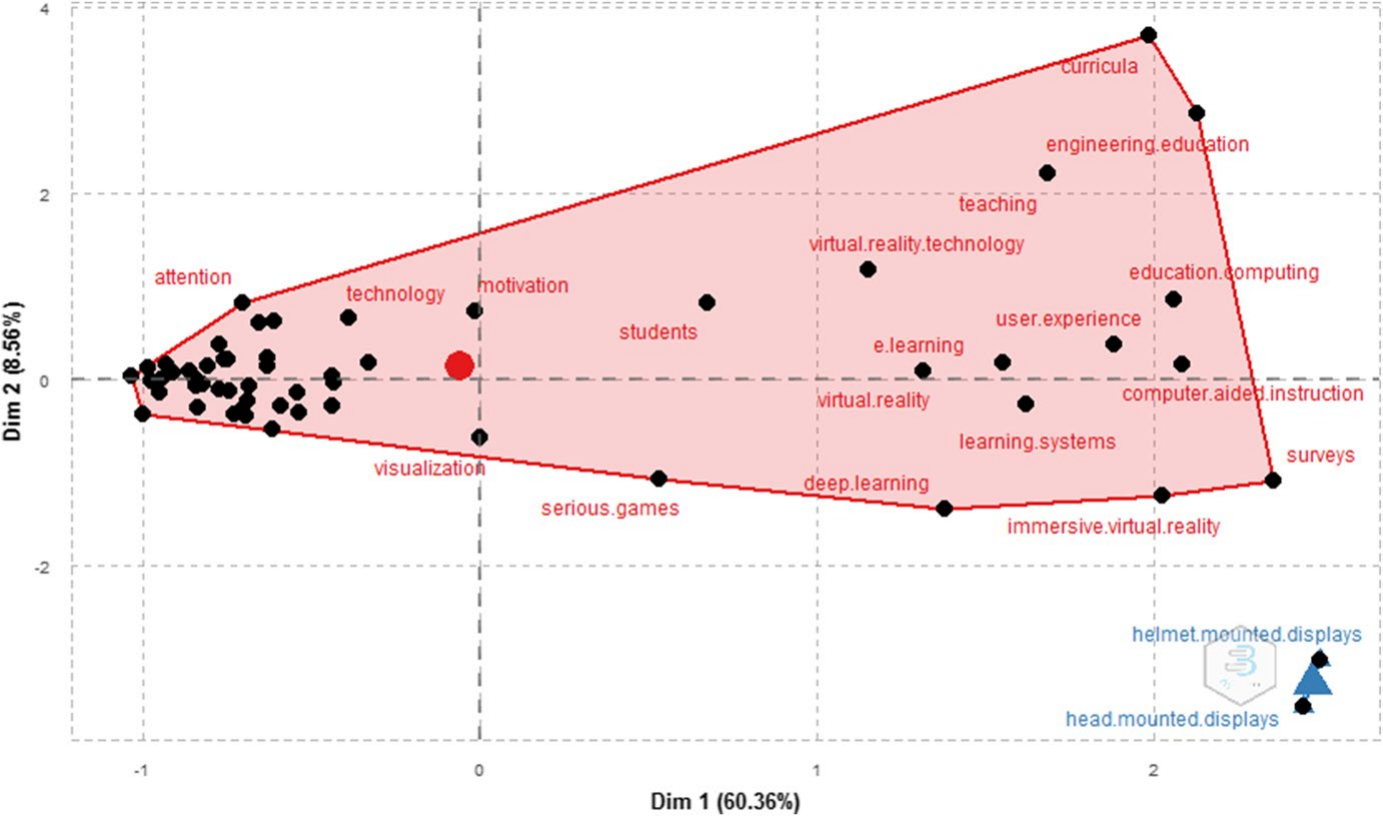
Factor map cluster analysis of high-frequency key words**.** Note: Factor analysis, keyword map, number of terms: 50, number of clusters: 2, label size: 12, number of words: 500

Figure 11 shows a two-dimensional graph formed by the topic words in Keywords Plus of the cited papers. A multiple correspondence analysis can be used to summarize big data with multiple variables in a low-dimensional space, creating a two-dimensional map where the words near the central point of the group have received a lot of attention in recent years and those near the edge are topics which have been used less in research or have been incorporated into other topics (Xie et al., 2020).

The first cluster covers words related to VR display devices which are used by users of virtual reality educational products. When it is not clear which device to use in a curriculum, the relevant constituent components of immersive technologies which differentiate their roles must be considered. An example is for the two common modes of virtual reality displays, head-mounted display (HMD) and desktop computer (DT) which may affect spatial learning (Srivastava et al., 2019). On the one hand desktop-based VR has higher installation costs, while mobile device-based virtual reality cannot produce the same environment quality due to the limited processing power. A result of the lower environment quality has, in some cases, caused higher rates of nausea and blurred vision (Moro et al., 2017).

A person can interact in an environment created with VR in a seemingly real or physical way by using special electronic equipment, such as a helmet with a screen inside it or gloves equipped with sensors (Katsioloudis et al., 2017). Jensen and Konradsen (2018) identify situations where HMDs are useful for cognitive skill acquisition, such as remembering and understanding spatial and visual information, psychomotor skills like head movement, visual or observational exploration and affective skills for emotional control and response to stressful or difficult situations.

The second group covers VR applied to education, which includes topics such as technologies that allow the visualization of situations, which receive more attention from students and motivate them. Examples of this type of technology are virtual communities, educational games, interactive learning environments, educational technologies that improve the teaching process, online learning, user experience, immersive learning, immersive virtual reality and deep learning. There is a wide variety of possibilities, most of which are immersive, using helmets, games or applications that provide an interactive learning experience for students.

Learning environments using animation and multimedia highlight a change in VR learning which is more immersive, simulating the real world with 3D models that provide an interactive environment and reinforce the feeling of immersion. Using this technology, educators combine theory and instruction methods that allow intelligent use of these environments (Huang et al., 2010). There are many ways to create these environments with equipment like VR helmets for experiential learning in a virtual space (Kwon, 2019) and new and improved environments, such as the PILE System that integrates video capture technology into the classroom where interaction is made through physical movements (Yang et al., 2010). As technology advances, better graphics and virtually animated actors or avatars can be used. These improve the applications by being more motivating and enjoyable, even though the applications become more complex which may prevent a novice learner from learning effectively (Kartiko et al., 2010).

VR applications enable potential learning. Authors such as Johnson-Glenberg, (2018) explore applications of educational theory which design classes using immersive virtual reality with two unique attributes of VR, which are making the student feel present in any given situation and to be able to use gestures and perform manipulations in three dimensions. For decades the primary interfaces of educational technology have been the mouse and keyboard, but now highly immersive environments can enhance learning and affect the way content is retained and encoded.

Games are useful in educational technology with many examples available. Some of these are used to train students in safety through role-playing and social interaction (Palos-Sanchez et al., 2018), which allows students to understand the causes of accidents and inspect risks in an immersive environment provided by the game (Le et al., 2015). Interaction was found to play an important role in understanding mathematics and geometry with problem solving. A whiteboard and a virtual tool were used to solve problems individually or in pairs. Group learning was found to be more effective, although the results of the groups were different as the difficulty of the problems were varied (Hwang & Hu, 2013).

Articles were found concerning online learning which shares digital content and technological tools for e-learning and virtual reality learning. One of these articles compares techniques such as email, attachments, shared use of Web interfaces and a VR engine which provides a virtual interface. The results indicate that users completed their workflow 50% faster with the VR option (Lampert et al., 2018). Another application that marked a change in e-learning is an innovative tool for young adults with mild cognitive impairments. It is an immersive virtual reality game called "In Your Eyes" that focuses on skills related to spatial perspective involving all five senses which shows that an immersive world can be an excellent training method (Freina et al., 2016).

#### Co-occurrence network mapping

**Fig. 12:**
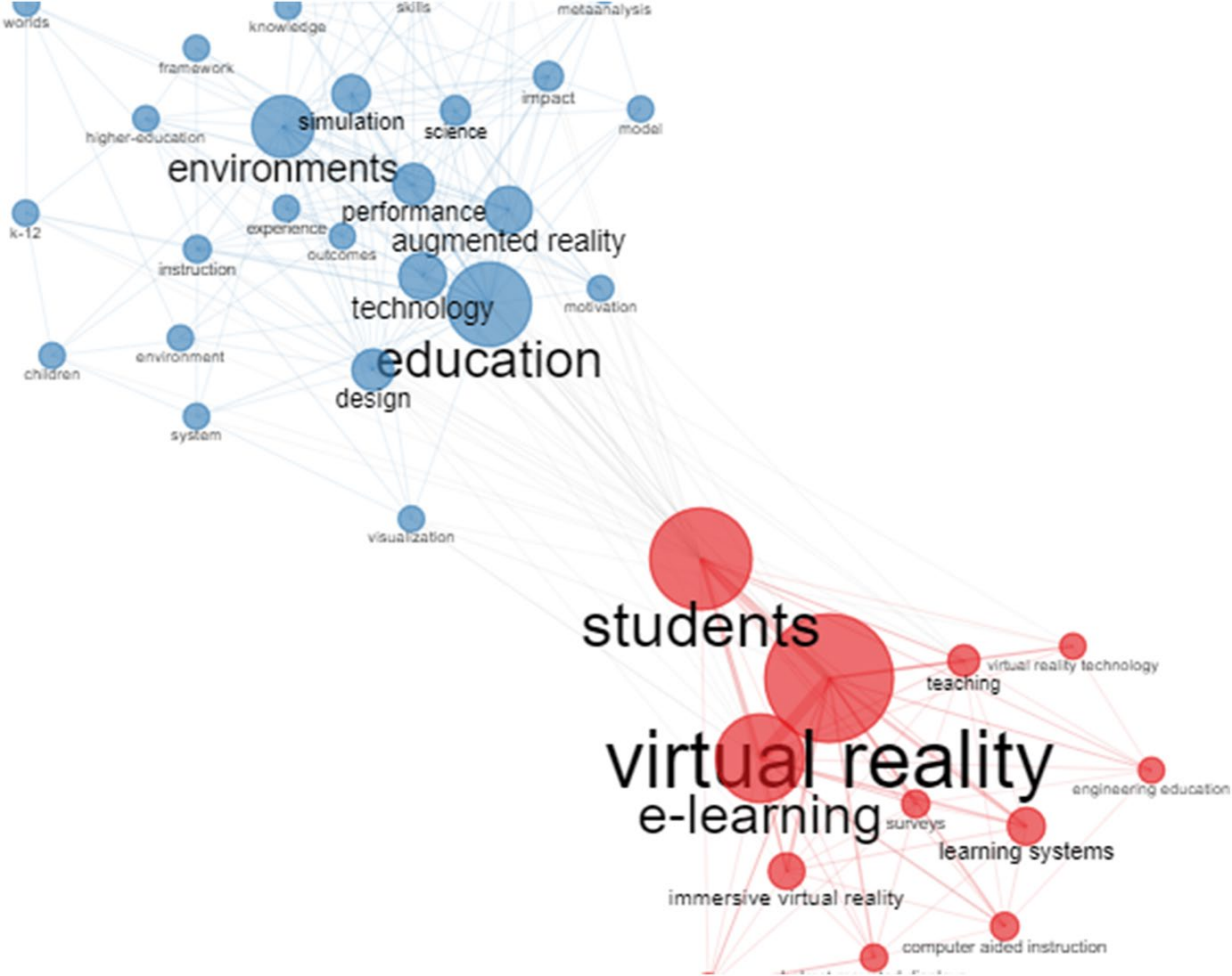
Keywords Plus co-occurrence network

Bibliometric mapping of the keywords used by the author was done to gain a thorough understanding of the conceptual structure. Apart from the Keyword Virtual Reality itself, it highlights those related to education, e-Learning and Students. The co-occurrence analysis is shown in Fig. 12.

Keyword co-occurrence analysis is an effective tool for understanding knowledge structures and research trends. This makes it easier to understand primary and secondary publications (Altınay Ozdemir & Goktas, 2021). In this figure, one should start by distinguishing nodes by their size. This represents the number of documents, while the line between two nodes represents a link between the two groups. A link means a co-occurrence between the two keywords (Guo et al., 2019). If the line is short the link is strong and vice versa.

In this bibliometric analysis we mainly distinguish the following keywords: 'virtual reality', 'e-learning' and 'students' in a first cluster. Each cluster represents a keyword and shows the most linked and repeated keywords in the publications. All clusters have a different colour. In Fig. 12 a distinction is made between the red colour for this cluster and the blue colour showing the following main keywords: 'Education', 'Technology', 'augmented reality', 'performance', 'simulation' and 'environments'. Because this bibliometric study found few papers, the number of co-occurrence links between keywords was not excessive. As Fig. 12 shows, two groups had a stronger relationship: 'Education Technology for simulation environments with augmented reality' and 'Virtual reality for e-learning systems'.

#### Productivity mapping of items by country

**Fig. 13:**
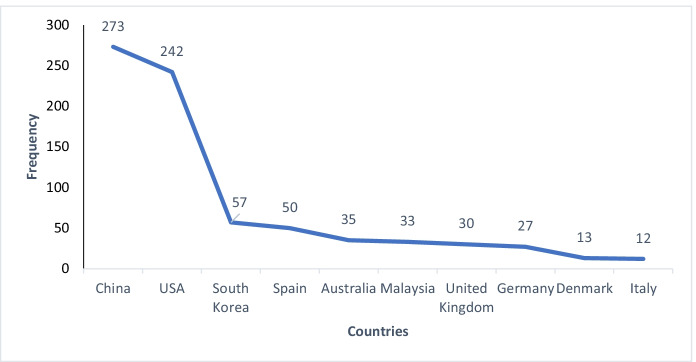
Productivity of items by country

The countries or regions with the highest document productivity in this study are the Republic of China with 273, United States of America with 242 documents, followed by South Korea with 57 and Spain with 50, as shown in Fig. 13. The high productivity of the United States is consistent with the bibliometric mapping for an analysis of studies on foreign language teaching in early childhood education by (Yilmaz et al., 2019) as well as the work of other authors (Hernández-Torrano & Kuzhabekova, 2019). China is a world power in VR technology and Chinese universities have been concerned to increase research in this field to meet the challenges posed by VR technologies. The main reason is that Chinese people are very prone to adopt emerging technologies, we can say that it is an important virtual reality market in the world.

**Fig. 14:**
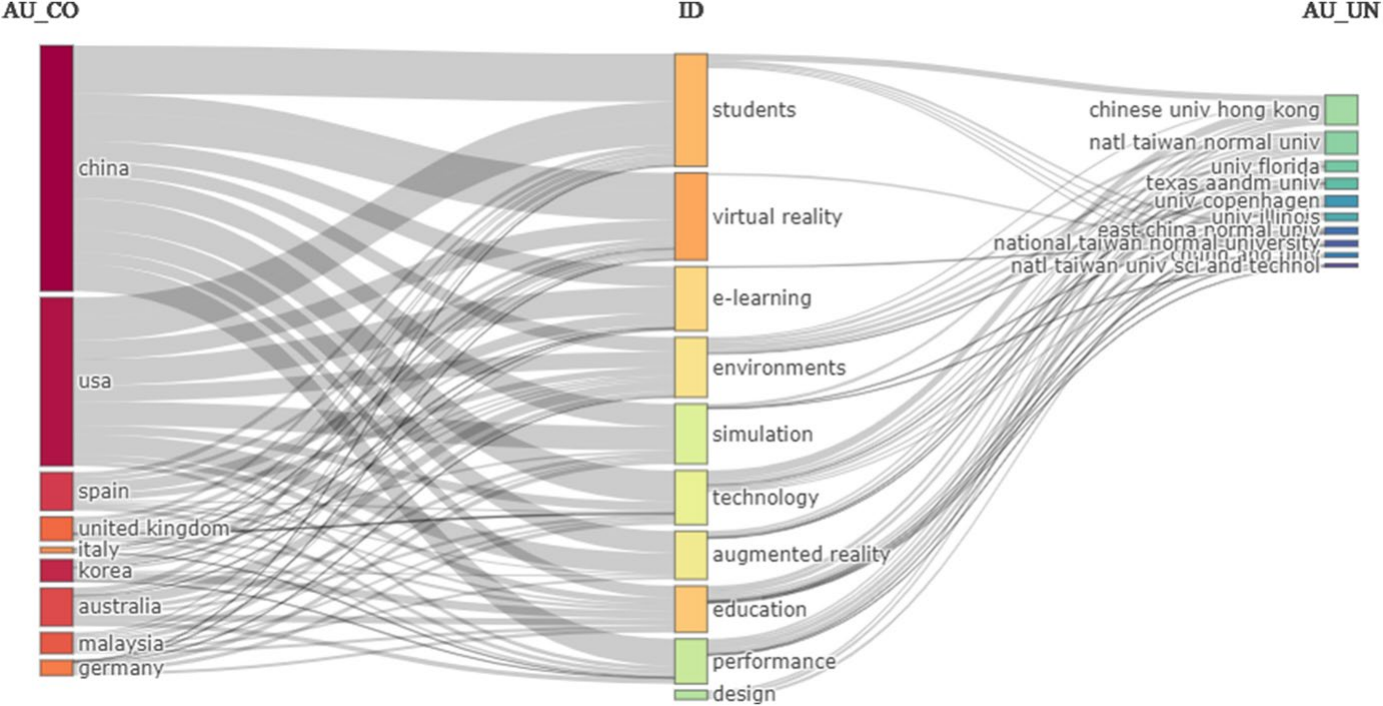
Three fields plot

In Fig. 14 we see how the main keywords, students, virtual reality, technology, e-learning, have a greater relationship with the countries of China and the USA, as well as the universities in the last column, which reflects a greater scientific production in topics related to technology applied to education.

**Fig. 15:**
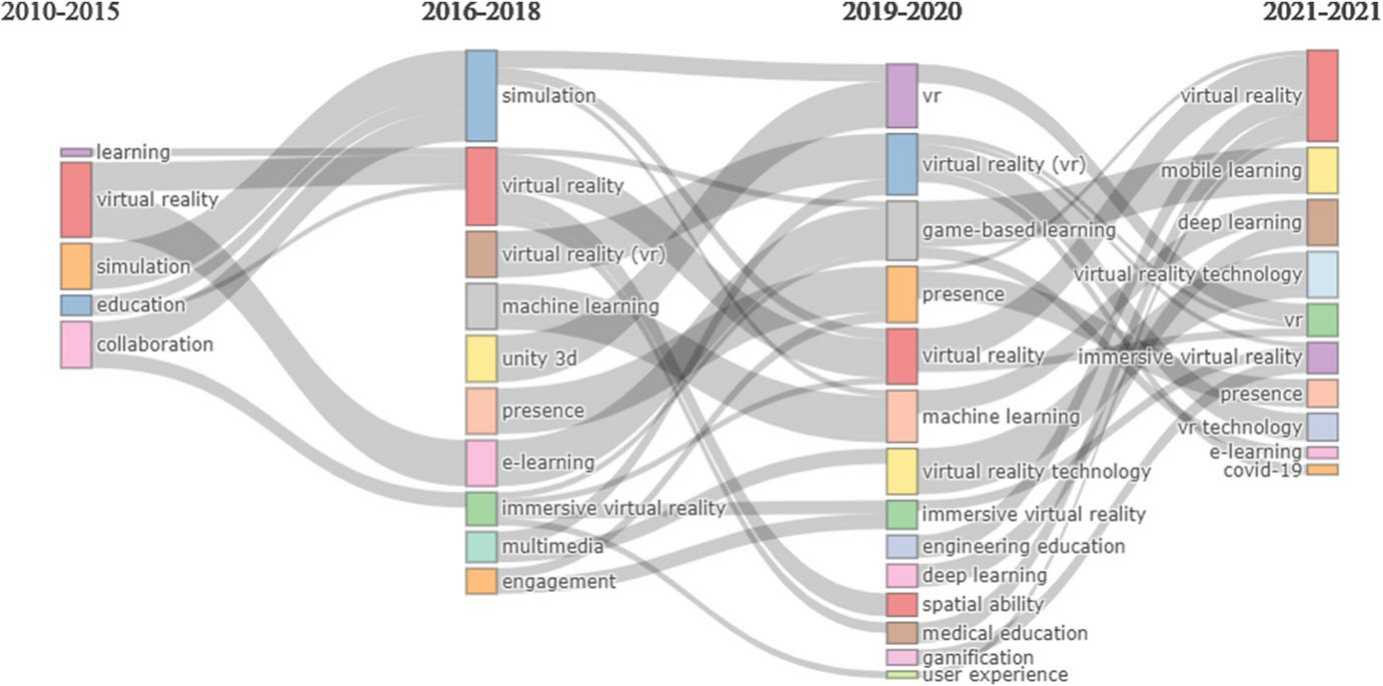
Thematic evolution

VR technology in the educational field is opening up space through e-learning, game-based learning to mobile learning, going from simulation, machine learning to Deep Learning, where immersive virtual reality is part of the topics present as shown in Fig. 15.

In Fig. 15 we can see the thematic evolution through a Sankey energy diffluence diagram, which is a specific type of flow diagram. In this paper, based on the Sankey diagram, we visualise the thematic evolution over time in the field of VR and Education research. This figure helps us to understand the temporal evolution of the conditions in which the different topics in the field of Virtual Reality applied to Education have been flowing. In this Fig. 15 we can clarify quantitative information such as thematic flow, direction of thematic flow and conversion relationships (Soundararajan et al., 2014).

#### Collaboration between countries mapping

**Fig. 16:**
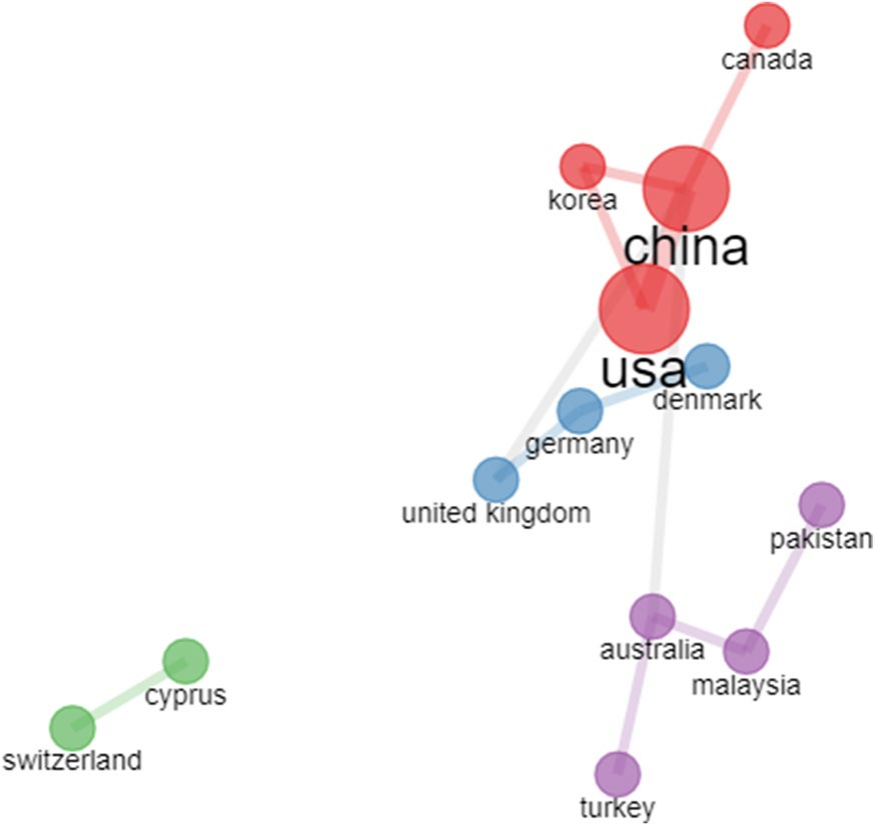
Collaboration between countries

This map gives an improved understanding of the social structure, not only of authors, but also of the countries to which they belong. Figure 16 shows collaborative relationships between China, USA, Korea, and Canada, as well as Germany, Denmark and the United Kingdom, along with others, the collaboration between the USA and China are the most important.

### Second stage analysis

This section briefly summarizes the most cited articles, and they are classified into categories, in the literature few authors provide us with a panoramic view of virtual reality technology applied to the educational field. In contrast to other authors (Zappatore et al. (2015) who perform an eminently quantitative approach in their analyses, this paper follows the line of Heradio et al. (2016) by providing a dual quantitative–qualitative approach. Thus, our analysis is not limited to counting articles, authors or journals, but describes and comments on the most relevant data for the RV community (Bardakci et al., 2022; Kushairi & Ahmi, 2021). The articles were listed in descending order according to the number of citations to find the main topics addressed and describe the documents that are considered the most important. All papers which had citations were classified and grouped into six categories: (a) papers about VR-based instruction and learning, (b) papers studying VR learning environments, (c) papers presenting the use of VR in different fields of knowledge, (d) papers describing learning processes that use VR applications, devices or games, (e) papers on research about learning processes using simulation, and (f) topics published during the Covid-19 pandemic.

#### VR-based instruction and learning

Among the most outstanding papers are the following: Merchant et al. (2014), Lee et al. (2010), Makransky and Lilleholt (2018) and Jensen and Konradsen, (2018). Jensen and Konradsen, (2018) seek to update knowledge on the use of head-mounted displays (HMD) in Education and training. The study identifies the acquisition of skills such as cognitive skills, i.e., remembering, understanding information, spatial and visual knowledge; as well as visual exploration or observation, among the most important are the affective skills related to control and emotional response to stressful or difficult situations. These learning tools enhance learning and are very useful in the educational field.

The most cited paper is Merchant et al. (2014). This work performs a meta-analysis that investigates the effectiveness of reality-based virtual instruction on learning outcomes. In order to do this, the authors researched the overall effect and impact of selected instructional design principles of VR technology-based instruction such as, games, simulation, virtual worlds, in higher education settings and as a result found that using games has a greater effect on learning than simulations and virtual worlds.

The study by Lee et al. (2010) examined how desktop VR (VR) enhances learning, finding that VR features have an indirect effect on learning. The learning experience was individually measured by psychological factors, such as presence, motivation, cognitive gains, control, and active learning, as well as reflective thinking which all affected the learning outcomes when using the desktop VR-based learning environment. Further research investigated how spatial ability and learning style enable instructional designers and VR software developers to improve learning effectiveness and therefore increase the amount the software is used. According to Makransky and Lilleholt (2018) much remains to be discovered about the impact and use of immersive VR in e-learning tools that impact students' emotional processes while learning.

Many authors investigate VR as a tool in learning processes. An example is Huang and Liaw, (2018) who explored how virtual reality technology actively focuses on the learner's interactive learning processes and attempts to reduce the gap between learner knowledge and real-life experience. Alfalah (2018) examined perceptions when using VR as a tool for education confirming that teachers and students are willing to use VR.

Allcoat and von Mühlenen (2018) assigned students into three groups who taught with different methods, 1) traditional book learning, 2) virtual reality learning and 3) video. The students were tested for their knowledge of the subject being taught before and after the classes, finding that participants in the virtual reality group showed better recall performance and more positive emotions than the other groups.

Hewawalpita et al. (2018) explored an improved configuration of massive open online courses. Two groups were used, one group were students who had already taken the traditional course and the second group started from scratch and were given virtual reality content. The results showed that the second group had significantly better performance and it was concluded that interactive learning content can be designed for the different learning needs of students.

Wang, (2018) proposed a distance learning virtual reality experiment with computers and VR technology and found practical reasons to promote the development of distance learning using computer.

In view of the works analyzed in the context of VR-based Instruction and Learning, we can say that the VR-based learning process is a useful tool for the educator, as it can replicate or complement traditional teaching methods. It is a fully effective concept even using basic forms, such as VR glasses or smartphones. This method is suitable for classroom teaching, distance learning, self-learning and other educational environments, and allows the simulation of scenarios that enrich teaching, even those dangerous experiments that cannot be reproduced in reality.

These papers conclude that it is essential to design useful and learner-friendly VR learning tasks and activities in order to improve learning outcomes. These activities should be adapted to learners with different learning styles and special abilities. Although this is a novel experience, the gap in these research models lies in the need for new longitudinal studies to verify whether improvements in teaching processes are maintained over time. It is necessary to identify differences in teaching processes, such as context, sample, duration, cultural background or learning programs with different content.

There is a need to deepen and explore the influence of virtual reality on the relationship between motivation and learning performance. That is, it is desirable to know whether students' disappointment can have a negative influence on their learning, using qualitative and quantitative methods in the study of prolonged periods.

#### VR learning environments

Geng et al. (2021) explore the pedagogical potential of Interactive Spherical Virtual reality based on video in geographic education, considering the perspective of teachers, they were given an introduction to this technology to know the acceptance, creation and experience, it was intended that teachers know the potential of this technology for teaching and learning purposes, the main concerns were the technological integration in pedagogy, they find that they need more professional development to design and refine this methodology. Perhaps it is more change adversity that is reflected in the need to ask for more training in the use of technology.

VR learning environments are explained by Huang et al. (2010) who indicate that there is a shift in learning from conventional multimedia to a more immersive, interactive, intuitive and exciting VR learning environment. It combines positive pedagogy and the use of technological innovations that are immersive and trigger the imagination of the learner.

Fowler, 2015 tried to give a more pedagogical description of adopting learning in three-dimensional (3-D) virtual learning environments (VLE) using a "design for learning" perspective that is useful for those who design learning activities in 3D VLEs, but considers that the risk of high-fidelity 3D VLEs is that using them to create virtual classrooms that "feel" and look like real classrooms means that they miss the opportunity to create pedagogically new and innovative learning environments.

Yang et al. (2010) investigated designing and developing a physically interactive learning environment. This was a PILE system that integrated VR video capture technology in a classroom. The group using the system showed a significant difference in pre-test and post-test knowledge. Makransky and Petersen (2019) believe that VR has the potential to enrich students' educational experiences. The authors investigated the affective and cognitive factors that play a role in learning when using desktop virtual reality simulation and concluded that learners can benefit from desktop virtual reality simulation in which emphasis is given to effective virtual reality features with a high level of usability.

The works analyzed emphasize that previous experiences with virtual reality in education have improved significantly. Although in the beginning they only used a mouse and keyboard as input devices, the benefits and educational effectiveness of 3D virtual learning and new virtual tools such as the PILE system, which allows students to interact with objects on the screen through physical movements, are gradually emerging.

Although technology has accompanied the teaching process exponentially in recent years, replacing traditional whiteboards with smart boards and VR elements, the gap in these research models lies in the need for new research is needed to explore the variables that may affect learning outcomes when using VR simulations. The described works suggest that it would be convenient to explore aspects such as duration, users' prior knowledge or dual cognitive/affective component.

The study of individual student differences, the long-term implications for knowledge acquisition, the frequent use of technology outside of teaching, the ease of use of different VR tools and the willingness of teachers to implement new VR-based utilities are also considered. This analysis evidences the importance of VR-based simulation processes, especially in areas of knowledge development that teachers deem necessary, allowing a balance between the cost and benefit of the experiences obtained.

#### Use of VR in various fields of knowledge

Osti et al. (2021) They seek to train construction workers using a novel VR system, this simulated a virtual training site, implemented a 3D training video with a VR head-mounted display, and compared it with a second group shown simple 2-D instructional video training, the first group presented better results in terms of retention, task performance, learning speed and participation. The practical application of VR as a teaching and learning tool is remarkable.

Schmidt and Glaser (2021) investigated the use of virtual reality by individuals with autism using 360-degree video modeling and headset-based virtual reality to investigate skills acquirement in adults on the autism spectrum in order to promote safety and the appropriate use of public transport. The results suggest a very positive learning experience and that the intervention is feasible and relevant for the unique needs of the target population.

Vélaz et al. (2014) studied the influence of interaction technology on the learning process when performing assembly tasks and learning processes using games and VR applications for industrial education. Sampaio et al. (2013) investigated the use of VR in civil engineering education by using it as a tool to create interactive applications as part of research work with students in which VR applications were developed for use in the construction industry.

A study by Eaves et al. (2011) determined the effects of two variations of real-time VR and feedback when learning a complex dance movement. Crocetta et al. (2018) presented and described a VR software package that helps in the rehabilitation of people living with disabilities. The findings of the study suggest that motor skills could be influenced differently depending on the environment and interface in which the software is used.

#### Learning with VR apps, devices or games

Chen and Hsu (2020) used a VR game-based English mobile learning application to investigate the effectiveness in English learning from a cognitive and psychological perspective, finding that interaction with the virtual reality application and the challenges of a game-based design allow students to enter the flow state easily and enhance their motivation to learn.

Authors M. Zhang et al. (2018) studied recent developments in game-based VR educational laboratories. According to the author there are several inherent disadvantages of VR that prevent its widespread deployment in the educational field such as unrealistic representation, lack of customization and flexibility, financial feasibility and the physical and psychological discomfort of users.

Sood and Singh (2018) considered that educational games for electroencephalography (EEG) can be widely used to improve the cognitive and learning skills of students. This can be achieved with the combination of VR and computing that provides accessible e-learning education worldwide. Psotka (2013) indicated that new technology such as VR and educational games can often disrupt established practices and are therefore considered disruptive technologies. The author believes however that they are appropriate for education and training today but have not been accepted in education due to changing social lifestyles.

#### Learning processes using simulation

Makransky et al. (2020) investigated the value of using immersive virtual reality (IVR) laboratory simulations in science education in two studies. The first study used an IVR laboratory safety simulation with pre- and post-test design. The second study compared the value of using IVR simulation and video simulation for learning the topic of DNA analysis. The results show that in both groups there were significant gains in self-efficacy and physical outcome expectations, but the increase in career aspirations and personal outcome expectations did not reach statistical significance.

Hsu et al. (2016) considered that visual simulation technologies have received considerable attention in learning. A vehicle driving simulation system was created to assist novice drivers in practicing their skills by considering various environmental driving factors that may be encountered while traveling. Dubovi et al. (2017) evaluated the effectiveness of VR learning simulation in pharmacology for higher education students requiring special skills to learn about medications and the procedure for administering them. The results revealed higher conceptual and procedural knowledge than with solely lecture-based learning.

#### Topics published during the Covid-19 pandemic

Wu et al. (2021) They use an immersive virtual reality approach based on video, they developed a landscape architecture VR learning system, due to the fact that during the Covid-19 pandemic the fields are closed, in addition, online education lacks the necessary scenarios for the courses taught during the pandemic, so better results and learning attitudes are achieved than students not subjected to this VR system. The importance of VR as a learning tool is evidenced in the face of the limitation of a real environment, here technology becomes an important ally.

Paszkiewicz et al. (2021) presented an educational process for Industry 4.0 that included the design, creation, implementation and evaluation of individual courses implemented in a virtual reality environment, identifying significant advantages and disadvantages of VR-based education. The development and implementation of appropriate courses in the virtual reality environment was found to reduce costs and increase the safety and efficiency of activities.

Yerden & Akkuş, (2020) examined the effects of the use of a Virtual Reality Supported Remote Access Laboratory (VRRALAB) system using remote access and virtual reality technologies on students' learning experience. The interactive use of a real device with a VR-supported remote access laboratory environment does not have any risks for novice users. The results indicate that remote access labs using virtual reality are likely to increase learning quality and student satisfaction levels.

Taçgın, (2020) investigated the characteristics of an immersive virtual reality learning environment (IVRLE) by evaluating perceived simulation effectiveness for student learning, attitude, and confidence by using gesture interaction to teach preoperative surgical procedures and concepts to undergraduate nursing students. Well-designed and targeted IVRLE was found to help to improve students' confidence in practical skills. Wang, (2020) applied virtual reality techniques in modular teaching to construct virtual simulation teaching resources and built two teaching modules that are visual, interactive, scalable, upgradable and optimizable. The results of the research suggest a new method of modular teaching and are a useful reference.

## Discussion

The results showed that the production of documents on VR in education has increased since 2015, possibly due to the increase in interest in virtual reality technology. There were important changes in the field in 2014 with the introduction of the Oculus Rift Frame. The interest in VR is reflected in the number of publications in 2018 and has been increasing with the changes in education systems due to the Covid-19 pandemic. The productivity in the last two years has been much higher than before.

The growth is then constant from 2015 onwards, increasing 10 times by 2020. There was a high number of publications in 2019 and a much higher number in 2020, possibly due to the new interest in VR and the impact of the Covid-19 pandemic. Higher production is expected for VR in Education in 2021. The recent growth is consistent with the results of the study on VR and motivation in the educational field (Soto et al., 2020) and with the work in the field of rehabilitation (Huang et al., 2016).

Figure 14 shows that China leads the number of publications with 273 articles, but interestingly the journals with the highest number of publications are the German International Journal of Emerging Technologies in Learning and Computers & Education from the UK. These journals also had the highest impact in the studied dataset. The difference of China as the leader of publications and the publishing journals may be due to the papers contained in the selection process. From the total of 298 journals and 718 papers that make up the dataset, there are 72 papers that were written by a single author. Most publications were written by an average of 2.95 authors, which means that from 1939 authors there are 1867 authors with papers with multiple authors. These papers are widely cited, with an average number of 10.03 citations per paper over a 10-year period and an annual average number of 2.49 citations per paper. Interestingly, most citations were in 2010 with an average annual number of 6.97 citations per paper, 2014 with 7.13 citations per paper and 2018 with 5.99. The data for the Covid-19 pandemic period, which started in 2020, is clearly shown in Fig. 4. The two most cited papers were (Merchant et al., 2014) with 574 citations in volume 70 of the Computers & Education journal and (Huang et al., 2010) with 321 citations in volume 55 of the same journal.

Figure 7 presents the authors with the highest impact after analyzing values of the h-index. In this study they were found to be Chen Y. and Jong M. both with an h-index value of 7. Figure 6 shows the authors Chen W., Chen Y. and Lee J., who are considered very productive because they have 8 publications.

Co-occurrence was used to identify the conceptual structure by analyzing the words in the Keywords Plus of cited articles. The words that appear most often were found to be virtual reality with 107 occurrences, students with 70, education with 66, learning with 58 occurrences, and environments with 44. All these words are closely related to the search topics although technology, performance, simulation, design and learning systems were also found. This information is shown in Fig. 10.

The words covering the topics in the Keywords Plus of the documents were analyzed by means of a factorial map and a cluster analysis, as shown in Fig. 9. Two dimensions were used, the first of which covers topics related to VR visualization devices by users of virtual reality educational products, and the second dimension covers VR uses in education. Words related to visualization, attention and motivation of students, the use of serious games, interactive learning environments, online learning and user experience to strengthen the use of VR in education were all found in this analysis stage.

Co-citation was used to analyze the intellectual structure. The authors of the documents were analyzed, and Lee was found to have a co-citation relationship Merchant, Dalgarno, Smith, Mikropoulos, Davis and Bouman, while a second group of co-citation authors was formed by Chen, Wang, Huang, Zhang, Chang, Yang and Lin. This information is shown in Fig. 11.

An inductive analysis found that the most cited papers were into the major categories of virtual reality-based instruction and learning, learning environments using VR, VR for teaching learning processes, instructional design and VR as a tool that enhances learning processes.

VR learning environments contain different systems where VR is applied in education, ranging from conventional multimedia to a physically interactive learning environment.

Research was found that investigated the use of VR in different fields of knowledge and various areas of education such as civil engineering, production lines and rehabilitation.

Learning processes incorporating the use of VR applications, devices or games are considered disruptive technologies that alter the usual teaching, cognitive and learning practices of students. However, the combination of VR with computing was seen to provide effective e-learning.

Articles were also found in the dataset about research using simulation models, for example driving vehicles, assembly lines, operations in the medical field. These simulation models used three-dimensional technology for the teaching process.

The research topics published during the Covid-19 pandemic examined the new trend in the field of education caused by the abrupt change from face-to-face teaching to virtual, online teaching. Research topics included deep learning and machine learning using artificial intelligence as alternatives to everyday teaching technologies and platforms. These included robot teleoperation, remote access laboratories, Virtual Reality-Based Cognitive Telerehabilitation Systems, Machine Learning Predictions, Immersive Virtual Reality learning environments, intelligent virtual reality technology, distance learning classrooms using machine learning and virtual reality, Virtual Reality-Interactive Classroom, Student Orientation in Distance Education Programs, Virtual Reality and BIM Methodology for Teaching–Learning Improvement, virtual reality-based gaming instruction and virtual reality for students' adaptive learning among others.

The six categories of the second analysis showed that improvements in VR learning processes have occurred in recent years and important advances have been made in the application and use of this technology. However, even though the engagement and motivation of students can be improved by using this technology, there still remains a lot to be discovered about the use of e-learning tools. VR learning environments have advanced significantly from multimedia to interactive environments with desktop and immersive VR processes, 360-degree tours and various applications and games. Education has generally adopted these changes slowly with most attention coming from the new generations of students. VR technology has existed for many years but has not been adopted as quickly as would be expected by educational centers, where it is seen as a disruptive process and is more commonly used by students for recreational purposes. The results of this study show that despite the multiple applications of VR in different fields of education, there is little evidence of incorporating them in teaching–learning processes. Educators have, however, quickly resorted to the use of technology platforms to teach because of the sudden change from face-to-face to virtual, online classes due to the Covid-19 pandemic. With the many available applications of VR in education and given that students have accepted the use of VR for recreational purposes, it can be incorporated as part of educational and training courses and demonstrations, as well as being useful for evaluating students.

Finally, the quality assessment of this research work was evaluated applying contrasted methods, following recommendations proposed by Ramey and Rao (2011) and Rao and Ramey (2011). Similarly, the quality of the research work was evaluated by incorporating bibliometric criteria, especially in the automation of the whole process, by Pulsiri and Vatananan-Thesenvitz (2018). External validity was contrasted with the criteria of dos Santos Rocha and Fantinato (2013) and Nguyen-Duc et al. (2015).

## Conclusions

Bibliometric analysis is a valuable tool that can be used to reveal the evolution of the articles contained in the dataset and answer research questions.

The conceptual structure shows that the most-used words are related to the search terms, although some words were found to be used with higher frequency. These were virtual reality, students, education, e learning, teaching, while the main topics investigated range from basic ones such as virtual reality and education to technology, teaching, visualization, student motivation and attention, e-learning, learning system, deep learning, and immersive virtual reality.

Recent publications show an increasing interest in VR, but rather than delving deeper into this technology other technological options are being explored with combinations of VR and other technology and systems. The pandemic improved our abilities to adapt and innovate with virtuality, along with teleworking and virtual teams (Garro-Abarca et al., 2020, 2021). There has been a lot of research into tools that allow educators to improve and even reinvent teaching processes. Virtual reality applications are interactive and immersive with telepresence and education might reconsider its opinion of this technology and take advantage of it to make classes more enjoyable with the new normality we are living.

One of the contributions of this study has been to confirm the progress of VR technology. In the future, educational centers will be able to solve many challenges, such as learning by experiencing and interacting with an environment, instead of passively receiving the information to be assimilated. VR favors the motivation and involvement of students and educational staff in general, in addition to increasing the speed of learning. b. The frontier and future of VR learning environments. On the other hand, the frontier and future of VR learning environments, the article predicts that the increasing diffusion of virtual reality learning environments forces to create limits. One of the limits is based on the capacity of these didactic tools to improve their effectiveness at a training level compared to other more traditional methods. In addition, other aspects such as user privacy and equal opportunities for all students must be taken into consideration.

Thus, the trends of type and use of VR in different fields of knowledge will be directed towards the inclusion of experiential VR models, adapted to each field of knowledge. The aim is to create learning vehicles that enhance the acquisition of knowledge by students in specific fields of knowledge. In this context, the training of teachers in the use of these technologies within a clear educational framework that is adaptable to the different academic disciplines will be increasingly important. In this sense, the future of VR in education will depend to a large extent on the motivation generated in students, and on being able to identify the necessary characteristics to achieve an adequate level of learning through VR. The push for VR-based education, brought about by the Covid-19 pandemic, is expected to make this style of learning one of the educational preferences in the future, especially for self-learning and teaching in certain areas where VR technology is more developed.

This study was limited to investigating the use of virtual reality technology in education. Articles dealing with the medical field were not selected as primary data sources because the focus of research was on the application of VR in teaching and learning processes in secondary and university education, as well as other applications in conferences or training processes. However, articles were included from multidisciplinary areas that include research in the medical field to broaden the spectrum of practical applications of this technology.

Suggestions for future bibliometric analyses include the evolution of other subject areas, using new search terms that allow other articles related to the field of education to be included for a broader analysis of the metadata and investigating other present and future lines of research.

## Data Availability

Data sets used and/or analyzed during the current study are available from the corresponding author under reasonable request.
